# Comparison of Answers between ChatGPT and Human Dieticians to Common Nutrition Questions

**DOI:** 10.1155/2023/5548684

**Published:** 2023-11-07

**Authors:** Daniel Kirk, Elise van Eijnatten, Guido Camps

**Affiliations:** ^1^Division of Human Nutrition and Health, Wageningen University & Research, Helix, Stippeneng 4, Wageningen 6708 WE, Netherlands; ^2^Department of Twin Research and Genetic Epidemiology, King's College London, St Thomas' Hospital Campus, 4th Floor South Wing Block D, Westminster Bridge Rd, London SE1 7EH, UK; ^3^OnePlanet Research Center, Plus Ultra II, Bronland 10, Wageningen 6708 WE, Netherlands

## Abstract

**Background:**

More people than ever seek nutrition information from online sources. The chatbot ChatGPT has seen staggering popularity since its inception and may become a resource for information in nutrition. However, the adequacy of ChatGPT to answer questions in the field of nutrition has not been investigated. Thus, the aim of this research was to investigate the competency of ChatGPT in answering common nutrition questions.

**Methods:**

Dieticians were asked to provide their most commonly asked nutrition questions and their own answers to them. We then asked the same questions to ChatGPT and sent both sets of answers to other dieticians (*N* = 18) or nutritionists and experts in the domain of each question (*N* = 9) to be graded based on scientific correctness, actionability, and comprehensibility. The grades were also averaged to give an overall score, and group means of the answers to each question were compared using permutation tests.

**Results:**

The overall grades for ChatGPT were higher than those from the dieticians for the overall scores in five of the eight questions we received. ChatGPT also had higher grades on five occasions for scientific correctness, four for actionability, and five for comprehensibility. In contrast, none of the answers from the dieticians had a higher average score than ChatGPT for any of the questions, both overall and for each of the grading components.

**Conclusions:**

Our results suggest that ChatGPT can be used to answer nutrition questions that are frequently asked to dieticians and provide encouraging support for the role of chatbots in offering nutrition support.

## 1. Introduction

Over the last few decades, developments have made advanced technologies commonplace in everyday life in the developed world, facilitated by the accessibility and convenience enabled by the Internet and smartphones. This has led to an increased usage of these technologies for the acquisition of knowledge that previously would have only been available from healthcare professionals [1, 2]. Now, recent developments in the realm of artificial intelligence (AI) are making this process even easier by offering chatbots, which make use of natural language processing to understand and respond to text input in a way that mirrors human speech or text [3]. The most noteworthy example of this has been OpenAI's ChatGPT, which is reported to be the first application to reach 100 million monthly users, making it the fastest-growing application ever [4]. ChatGPT's apparent proficiency in a variety of fields makes it an attractive option for gaining a surface-level understanding of specific questions within a topic without having to search through many pages of search engine results.

Nutrition is one such field that has been particularly exposed to knowledge-seeking behaviour on the Internet. Studies show that a significant proportion of the population relies on the Internet for receiving nutritional information and that the majority of all sources of nutritional information in the developed world come from Internet-based sources, vastly more than books, journals, or other people [3, 5]. Whilst most of these come from single websites accessed directly or through search engines, using chatbots like ChatGPT to answer nutrition questions has advantages such as synthesizing information from many different sources, thus giving a more balanced perspective, and providing information all in one place rather than dispersed across multiple webpages. Additionally, the ability to converse with ChatGPT opens the possibility for discourse, which can allow clarification and question-asking to facilitate learning, which is not always possible in other web-based platforms.

Although far from extensively studied, chatbots have been researched in a nutrition setting in recent years. Specialized chatbots have been developed for adolescents, the elderly, and for those wanting to manage chronic diseases [6–8]. Since these and similar tools are designed specifically for conversations related to nutrition, it might be expected that these approaches would be superior to a general purpose application like ChatGPT. However, results in the studies were mixed, and the fact that ChatGPT has been trained on a much larger volume of data means that this is not necessarily the case, motivating the need for research in this area.

In fact, the challenges and opportunities of using ChatGPT in a variety of settings (both clinical and otherwise) were recently described [9]. Indeed, theoretical advantages such as those described above were included in support of the use of ChatGPT for answering nutrition questions. Some prompts were also asked by the authors of the paper about a diet suitable for someone with diabetes, and the responses were judged to be accurate by the authors, although the quality of the responses was not formally tested. Other studies have used ChatGPT to make diet plans for making dietary plans for specific dietary patterns, weight management, and food allergies [10–12], although, again, in none of these cases was the capacity of ChatGPT to answer common nutrition questions investigated, and studies tend to lack a formal assessment of the quality of the responses from ChatGPT from experts blinded to the goals of the study.

Chatbots are also expected to play a role in the growing field of precision nutrition, which aims to provide tailored nutritional recommendations based on personal information [13, 14]. Since it is infeasible to provide 24/7 access to a human expert to answer nutrition-orientated questions, it is likely that chatbots will fill this role and support application users looking to improve their health through dietary changes. Indeed, some research has already investigated using natural language processing tools to support application users with a specific goal, such as weight loss or chronic disease management [15–17]. However, ChatGPT's impressive natural language processing abilities have attracted an impressive number of users, and this widespread use means it will likely be trusted to answer nutrition questions despite not being specially developed to do so.

This makes it interesting and important to know if it is capable of adequately answering commonly asked nutrition questions. Successfully doing so prevents the need for nutrition laymen to search for specialized applications that may come with service fees and instead take advantage of the free-to-use, highly accessible, general-purpose ChatGPT offered by OpenAI. Conversely, if ChatGPT is unable to answer such questions to the standard of dietary experts, this too warrants attention since users of the application should be made aware that the information they receive from it may be unreliable.

For these reasons, we sought to investigate how answers from ChatGPT compare to answers from human experts. We asked dieticians to list their most frequently asked questions and then answer them, before asking these same questions to ChatGPT. Across eight questions, the answers from ChatGPT outperformed those from dieticians, both overall and across every individual grading component. Our results provide valuable insights into the credibility of answers to commonly asked nutrition questions by the world's most used natural language processing application and can be used to inform the development of future generations of chatbots, particularly those tailored towards offering nutritional support.

## 2. Methodology

Our objective was to investigate the capabilities of ChatGPT in answering frequently asked nutrition questions. Registered Dieticians were asked to provide nutrition questions that they most commonly received, as well as provide their professional responses to these questions.

We reached out to Registered Dieticians across the Netherlands through contacts within the university and networks of Registered Dieticians. Any questions related to nutrition reported by the dieticians would be included as long as they did not fall under the exclusion criteria. Exclusion criteria included questions that refer to very specific disease states or that afflict only a small portion of the population.

In total, we received 20 questions from 7 dieticians. The dieticians had a median age of 31, a mean age of 39, and ranged from 29 to 65. All of the dieticians were female and lived and practiced in the Netherlands, either in private practices or medical centers. Of the 20 questions, 12 were excluded for containing information specific to medical conditions. The remaining 8 questions from three different dieticians thus constituted the final questions used in the study (Table 1), and these questions were then asked to ChatGPT (Version 3.0) in their original form. The answers to each of the questions from the dieticians and ChatGPT can be seen in English and Dutch in Table S2.

Although Question 7 (see Table 1) was given to us as a statement rather than a question, we decided to leave unaltered. The reason for this is that ChatGPT is capable of inferring meaning from text, and thus we hypothesized that it would still be able to respond as if it was a question. We preferred first attempting this rather than changing the question as it was delivered to us, which could risk introducing bias.

The questions were only asked once and the answer was recorded. Since the questions and answers (Q&As) were in Dutch, we requested that ChatGPT answered the questions in both Dutch and English. In two cases, modifications were necessary to remove one part in two questions that could have compromised blinding in the experiment, but the content and overall meaning of the answer were unaltered. These instances can be seen in Table S1. The lengths of the answers were comparable between the dieticians and ChatGPT. None of the dieticians was made aware of the goal of the study or the involvement of ChatGPT in order to prevent any influence of knowing that their answers would be compared to an artificial intelligence. The study took place from February to June, 2023.

The answers to each of the questions were then graded by other dieticians and experts in the field of the questions. A grading system was designed which could be used by multiple adjudicators and allow mean scores to be calculated and compared. Human dieticians and nutritionists must keep in mind key points when delivering nutritional information to their receivers, which the literature reports as including being a skilled listener and reader of emotions, making recommendations that the receiver can act on, tailoring recommendations in a personalized way, the client-dietician relationship, and communicating accurate scientific evidence in a comprehensible way, amongst others [18, 19]. Naturally, some of these do not apply when advice is administered via a machine anonymously; however, others constitute important aspects of delivering high-quality answers to questions in nutrition.

Based on these literature findings and the combined expertise of their authors in dietetics and nutrition counselling, three components by which to grade the answers to the questions in our study were selected: “Scientific correctness,” capturing how accurately each answer reflects the current state of knowledge in the scientific domain to which the question belongs; “Comprehensibility,” reflecting how well the answer could be expected to be understood by the layman; and “Actionability,” the degree to which the answers to the questions contain information that is useful and can be acted upon by the hypothetical layman asking the question. The rubric explaining how each component should be scored that was sent to the graders is presented in Table 2. Each component could be graded from 0 to 10, with 0 reflecting the worst score possible and 10 reflecting a perfect score. The overall score was obtained by averaging the scores of the other components.

To further reduce bias in our methodology, we recruited experts in the field of each question and other dieticians (i.e., not those who also provided the questions) to grade the answers. Each set of answers was graded by 27 graders, which were split according to the nature of the components. One of the three components, scientific correctness, was concerned with specific domain knowledge, and therefore 9 of the 27 graders for each question were domain experts in the field of the question. Experts were recruited from universities or research institutes and were required to have worked with the question topic in their research. The other two components were more generally related to the transmission of information, and thus the remaining 18 graders were dieticians or nutritionists. As with the dieticians providing the Q&As, graders were blinded to the true goals of the study and the involvement of ChatGPT.

### 2.1. Ethical Approval

All participants agreed to have their contributions used for scientific research and shared their contributions voluntarily and were free to withdraw at any time; there was no financial incentive. All participants were informed that their participation would be anonymous and that personal data would not be shared beyond the authors. The study was carried out in compliance with ethical standards and regulations of Wageningen University.

### 2.2. Statistical Analysis

A score of 0–10 was provided for each answer per question by each grader, allowing the evaluation of the answers as a whole and across each grading criterion. The statistical significance of group differences was derived using permutation tests with the function perm.test from the package jmuOutlier in R software [20] with the test statistical set to mean. For both the overall score and the scores of the components for each question, *p* values were approximated from 100 000 simulations to gauge the strength of evidence of a difference between the groups. The study was registered and is described in more detail on Open Science Framework [21]. The only modification involved using nonparametric tests (permutation tests) instead of *t*-tests in light of the statistical nature of the data obtained.

## 3. Results

**Figure 1:**
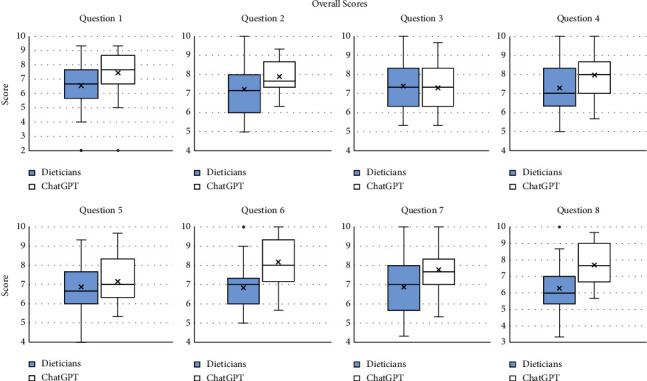
Results of the overall grades to the answers of the questions by the dieticians (blue, left) and ChatGPT (white, right). The upper and lower bounds of the boxplots present the median of the upper and lower half of the data, respectively, and the black line inside the box represents the median of the data. The upper and lower lines beyond the box (whiskers) are the highest and lowest values, respectively, excluding outliers. The cross is the mean of each set of grades.

All of the grades for each question can be seen in Table S3. Key summary statistics for the overall scores for the answers to each question can be seen in Table 3 and the corresponding boxplots in Figure 1. There was strong evidence that the mean overall grades for ChatGPT were significantly higher for five of the questions, namely, Question 1 (7.44 for ChatGPT vs. 6.56 for the dieticians), Question 2 (7.90 for ChatGPT vs. 7.23 for the dieticians), and Question 6 (8.17 for ChatGPT vs. 6.84 for the dieticians), Question 7 (7.78 for ChatGPT vs. 6.87 for the dieticians), and Question 8 (7.69 for ChatGPT vs. 6.28 for the dieticians). The scores of ChatGPT also tended to be higher for Question 4, although there was less evidence for a group difference than in the previous questions. Scores for Questions 3 and 5 were similar between the groups.

**Figure 2:**
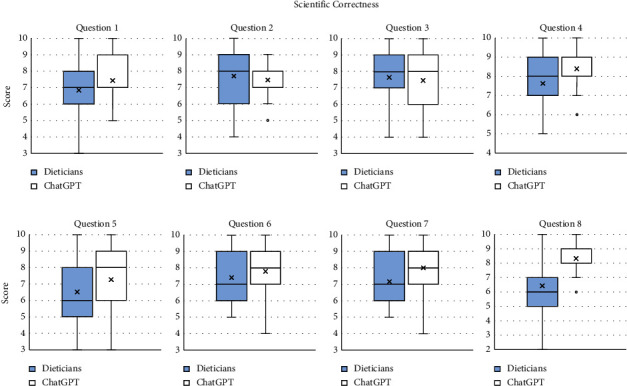
Grades for the component “Scientific correctness” for each question for the dieticians and ChatGPT.

**Figure 3:**
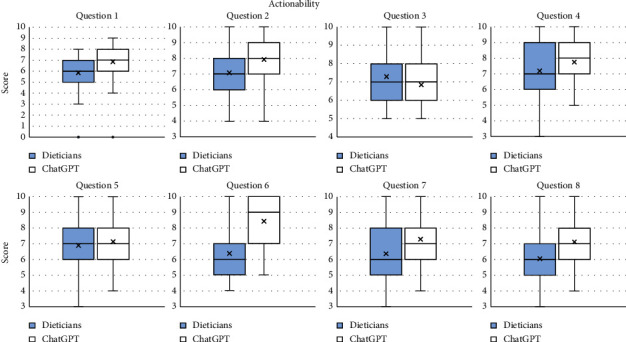
Grades for the component “Actionability” for each question for the dieticians and ChatGPT.

**Figure 4:**
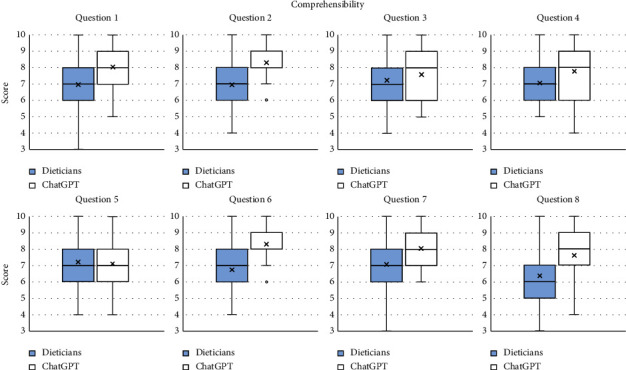
Grades for the component “Comprehensibility” for each question for the dieticians and ChatGPT.

Figures 2–4 show boxplots of the grades for the individual components, and tables of their summary statistics can be seen in Tables S4–S6. The mean scores for scientific correctness were higher for ChatGPT in five of the questions, namely, Question 1 (7.44 for ChatGPT vs. 6.85 for the dieticians), Question 4 (8.39 for ChatGPT vs. 7.63 for the dieticians), Question 5 (7.26 for ChatGPT vs. 6.52 for the dieticians), Question 7 (8.00 for ChatGPT vs. 7.17 for the dieticians), and Question 8 (8.33 for ChatGPT vs. 6.43 for the dieticians). No differences were observed for the remaining questions.

The mean scores for actionability were higher for ChatGPT on four occasions, namely, Question 1 (6.85 for ChatGPT vs. 5.85 for the dieticians), Question 2 (7.93 for ChatGPT vs. 7.07 for the dieticians), Question 6 (8.43 for ChatGPT vs. 6.37 for the dieticians), and Question 8 (7.11 for ChatGPT vs. 6.06 for the dieticians), and there was a trend towards higher scores for ChatGPT in Question 7 (7.30 for ChatGPT vs. 6.37 for the dieticians). There were no clear differences for the remaining questions.

Finally, grades were higher for ChatGPT on five occasions in the comprehensibility component, namely, Question 1 (8.04 for ChatGPT vs. 6.96 for the dieticians), Question 2 (8.30 for ChatGPT vs. 6.94 for the dieticians), Question 6 (8.30 for ChatGPT vs. 6.74 for the dieticians), Question 7 (8.04 for ChatGPT vs. 7.07 for the dieticians), and Question 8 (7.61 for ChatGPT vs. 6.35 for the dieticians). No other differences were found for the remaining questions.

Neither the overall grades nor any of the individual grades per component were meaningfully higher for the dieticians on any of the questions. This can be seen in Table 4, which also may suggest that the key drivers of differences in the overall scores were actionability and comprehensibility, since they were higher on most occasions when the overall scores were also higher. On questions 2 and 6, scientific correctness was not different between the groups, yet overall scores remained higher and actionability and comprehensibility were also higher. Additionally, on all occasions when both actionability and comprehensibility were not different, overall scores also did not differ, except for in question 4 which tended towards being higher. However, this hypothesis would have to be tested on a larger number of questions before concrete conclusions can be drawn. Sensitivity analysis using the median (instead of the mean) as the test statistic for the group differences was also performed. These results can be seen in Table S7, but the outcome of the results was unchanged. Overall, these results provide compelling evidence that ChatGPT is better able to answer commonly asked nutrition questions than human dieticians.

## 4. Discussion

This is the first study to investigate the ability of ChatGPT to answer common nutrition questions. There was strong evidence of group differences in favour of ChatGPT, as evidenced by higher scores in five of the eight questions for the overall grades, whereas the dieticians did not have higher overall scores for any of the questions.

In addition to averaging the scores of the grading components to give an overall grade, we hypothesized that analyzing the performance of the individual components separately might have revealed how performance differed between ChatGPT and the dieticians. However, no notable differences could be observed in the analysis of the individual components vs. the overall scores, and again ChatGPT had higher average scores for scientific correctness, actionability, and comprehensibility in five, four, and five of the questions, respectively. Strikingly, the average grades of the dieticians were not higher for any of the questions for each individual grading component. Hence, these results suggest that ChatGPT was generally better at answering nutrition questions than human dieticians. Additionally, whilst the dieticians who provided the questions and answers were Dutch, many of the graders were from diverse countries and backgrounds, and we have no reason to believe that the questions, answers, or results would not generalize outside of the Netherlands.

Whilst the potential of ChatGPT for answering nutrition questions has not been investigated, chatbots have been experimented with in recent years in nutrition as a means of offering nutrition support [6–10]. These studies show mixed results with regard to the efficacy of chatbots in achieving their objectives; however, many of these applications of chatbots are dealing with specific domains within nutrition, such as supporting chronic diseases, and are limited in their application. Indeed, to the best of our knowledge, none have access to the volume of data that ChatGPT has, and, in the opinions of the authors, none can match ChatGPT's level of conversational sophistication. Combined with the accessibility and popularity of ChatGPT, these points mean that, despite not being a specialized nutrition chatbot, it could potentially be used by more knowledge seekers for answering nutrition questions than most, if not all, existing chatbots designed for this purpose, further motivating the importance of researching its properties. Additionally, the possibility to use ChatGPT to answer common nutrition questions poses other advantages, such as being easy and convenient to use and able to give answers instantly and at any moment. Furthermore, in contrast to traditional web searches, ChatGPT can engage in multiple rounds of conversation, which can allow users to ask for clarification on doubts or elaboration of certain points in much the same way that a human expert would, which also provides learning opportunities that can enhance nutritional literacy. Finally, ChatGPT is also virtually free; hence, in light of these results, ChatGPT could be of significant utility for individuals who cannot afford nutrition counselling and thus may also be able to contribute towards overcoming nutritional disorders associated with a lower socioeconomic status, such as obesity [22].

In a more clinical setting, AI-powered chatbots could change the work landscape of dieticians and their relationship with their clients, as was observed in a study of physicians in a healthcare field that has similarities to that of dieticians [23]. It has been reported that physicians see the added value of chatbots in healthcare, depending on the logistics and their specific roles [24]. Translating these findings to the dietician's situation, chatbots can do the work of routine tasks, such as answering common nutritional questions and lowering the workload, leaving more time for complex tasks and personal interactions with clients or patients, which may accompany a shift in the role of the dietician away from providing information and more towards coaching. Additionally, it is possible that chatbots may lead to the empowerment of users and increase their involvement in their own health [25, 26]. During these early days of this development, dietitians should take charge and think both individually and as an association on how to adapt to the developments in their field and how to guide these in the right direction.

Although not seen in the results of our study, there are some important issues to consider in using ChatGPT for the acquisition of knowledge. Firstly, ChatGPT provides no confidence about the validity of its own answers. Whilst ChatGPT scored highly in scientific correctness across all questions, it can get things wrong, though this is unbeknownst to itself or the user since there is no indication of the confidence of the answers that it provides. Secondly, a chatbot relies on the user to provide relevant information. Accurate and tailored dietary guidance often requires consideration of specific medical conditions, allergies, or lifestyle factors. Users may lack knowledge regarding the essential details required to deliver these accurate dietary recommendations. Additionally, despite being trained on a large amount of data, the data that ChatGPT was trained on are only available up until September 2021 [27]. Whilst this is less of a problem for fundamental nutrition questions, it prohibits the incorporation of the newest findings and thus lags behind the current scientific state. Similarly, non-digital texts or those behind a paywall were not available for training, meaning this knowledge was not incorporated. Finally, there may be circumstances in which the divulgence of personal information may improve the quality of answers from chatbots like ChatGPT; in this regard, it is crucial to consider privacy and security issues since information may have serious consequences for users of the app [28].

Some important limitations should also be considered when interpreting the results of this study. The answers to the questions were all in the range of around 100–300 words. Whilst this did not feel limiting to the dieticians that submitted the questions, it can also be argued that the topics of the questions could not be adequately answered in such a short space due to the nuances inherent to them. However, both parties—ChatGPT and the dieticians—had the same restrictions imposed, and thus the results are those of a level playing field. Additionally, it is important to contextualize the findings, and in this sense, when the questions require answers that leave out complexities and provide a simple overview that tackles the essence of the question, ChatGPT appears to be superior to human dieticians.

The questions we received were loosely categorized into domains within nutrition. Only the question themes “Weight loss” and “Carbohydrates” appeared multiple times, and our sample size of eight questions could not permit an investigation of how ChatGPT performance might differ across subject areas. However, this should be considered in future similar research since the capabilities of ChatGPT appear to be inconsistent across different tasks, meaning its nutrition-specific subject strengths may also vary. This would be valuable to know as it could be taken into account when gauging the reliability of the answers provided by ChatGPT with regard to a specific question.

We blinded the dieticians to the aims of the study. Whilst this was done to avoid influencing the Q&As that they provided us with, it should also be admitted that dieticians might have been more careful with their answers and have taken their task more seriously in a professional setting with a client, and so the grades for the answers from the dieticians might be an underestimation of what their true value would be. Additionally, since the analyzed questions were based on suggestions of a random sampling of dieticians, an avenue of future research could be to use the grounded theory approach [29] based on interviews with a larger sample of dieticians to create a list of central issues in dietary sciences to produce a more substantiated list of questions to confirm these results. Finally, the criteria we used to grade the answers aimed to include those most important for answers to nutrition questions. However, this is somewhat subjective, and the redefinition of our criteria or the introduction of other components could alter the outcome of the results.

Overall, these findings may signify important differences in the way that people access nutrition information in the future. ChatGPT is virtually free, accessible at any time, and provides answers almost instantly, which are all advantages over human nutrition experts. Investigating the ability of ChatGPT and similar applications to offer nutrition and health support is imperative given the current trajectory of the use of chatbots in everyday life.

## 5. Conclusion

We have shown that the general-purpose chatbot ChatGPT is at least as good as human dieticians at answering common nutrition questions that might be asked to a nutrition expert. Future work should investigate the potential of chatbots to provide information to educate and improve the dietary habits of users.

## Figures and Tables

**Table 1:** The most frequently asked questions reported by the dieticians categorized by theme.

	Question	Question theme
1	Can I use intermittent fasting to lose weight more quickly?	Weight loss
2	How can I eat tastily whilst using less salt?	Taste
3	Are the sugars in fruit bad to consume?	Carbohydrates
4	Are carbohydrates bad?	Carbohydrates
5	Is it better to swap sugar for honey? Or sweeteners?	Carbohydrates
6	What is a healthy snack?	Healthy nutrition
7	I've tried many diets in the last few years. I lose weight, but then it comes back.	Weight loss
8	Is vitamin/mineral supplementation necessary?	Supplementation

**Table 2:** The explanations of each of the grading criteria sent to the graders.

Grading component	Description
Scientific correctness	How accurately each answer reflects the current state of knowledge in the scientific domain to which the question belongs. The requested word count of the answers (100–300 words) and the natural limitations on detailed explanation and nuance this imposes should be kept in mind when grading scientific correctness. The target audience of the layman and their expected level of scientific knowledge and nutritional understanding should also be kept in mind.
Comprehensibility	How well the answer could be expected to be understood by the layman. Comprehensibility should pertain mostly to the content of the answer; however, if grammatical errors hinder comprehensibility, then this may also be considered.
Actionability	The degree to which the answers to the questions contain information that is useful and can be acted upon by the hypothetical layman asking the question. For example, whilst bariatric surgery may represent an effective weight loss strategy for morbidly obese individuals, this would not be a helpful suggestion for someone with a BMI of 27 looking to lose a little weight. Hence, such an answer would score poorly on this component.

**Table 3:** Key summary statistics of the grades for the overall scores.

Summary statistics for average overall scores						
Question		Mean	Median	Interquartile range	Minimum	Maximum
1	Dietician	6.56	6.67	1.67	2.00	9.33
1	ChatGPT	7.44	7.67	2.00	2.00	9.33
2	Dietician	7.23	7.17	1.92	5.00	10.00
2	ChatGPT	7.90	7.67	1.33	6.33	9.33
3	Dietician	7.40	7.33	1.83	5.33	10.00
3	ChatGPT	7.30	7.33	2.00	5.33	9.67
4	Dietician	7.29	7.00	1.75	5.00	10.00
4	ChatGPT	7.97	8.00	1.50	5.67	10.00
5	Dietician	6.88	6.67	1.67	4.00	9.33
5	ChatGPT	7.17	7.00	1.83	5.33	9.67
6	Dietician	6.84	7.00	1.33	5.00	10.00
6	ChatGPT	8.17	8.00	1.92	5.67	10.00
7	Dietician	6.87	7.00	2.17	4.33	10.00
7	ChatGPT	7.78	7.67	1.33	5.33	10.00
8	Dietician	6.28	6.00	1.67	3.33	10.00
8	ChatGPT	7.69	7.67	2.00	5.67	9.67

**Table 4:** The grading components are listed against each question and a color scheme is used to represent occasions in which ChatGPT scores higher (green) or marginally higher (orange) than the dieticians.

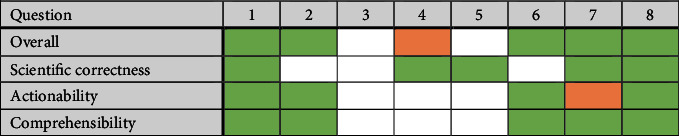

Since the dieticians did not score more highly on any occasion, there is no color to represent this. White represents no difference between the scores.

## Data Availability

Part of the data will be made available, namely, the answer grades and the Q&As. These are provided in the supplementary materials.

## Supplementary Materials

Supplementary Materials Table S1: the two answers from ChatGPT that were modified before being sent for grading in their original form and after modification. Table S2: answers to each question from the dieticians and ChatGPT in Dutch and English. Table S3: the grade of each grading component and the average overall grade for the answer to every question for both ChatGPT and the dieticians. Table S4: summary statistics for the grades of the component scientific correctness. Table S5: summary statistics for the grades of the component actionability. Table S6: summary statistics for the grades of the component comprehensibility. Table S7: the *p* values of the permutation simulations of the test statistic with the mean and the median.
