# Supplementary material for: Comparison of Answers between ChatGPT and Human Dieticians to Common Nutrition Questions
Source: J Nutr Metab. 2023 Nov 7;2023:5548684. doi: 10.1155/2023/5548684 (PMC10645493; doi:10.1155/2023/5548684)
Supplement: Supplementary Materials — Table S1: the two answers from ChatGPT that were modified before being sent for grading in their original form and after modification. Table S2: answers to each question from the dieticians and ChatGPT in Dutch and English. Table S3: the grade of each grading component and the average overall grade for the answer to every question for both ChatGPT and the dieticians. Table S4: summary statistics for the grades of the component scientific correctness. Table S5: summary statistics for the grades of the component actionability. Table S6: summary statistics for the grades of the component comprehensibility. Table S7: the p values of the permutation simulations of the test statistic with the mean and the median. [file 5548684.f1.zip › Table S1.docx]

Table S1: The two answers from ChatGPT that were modified before being sent for grading. The reason on both occasions was due to the fact that they both make a referral to a health care professional, and only this part of the answer was removed.

|  | |
| --- | --- |
|  | **Question 1** |
| Original Answer | Intermittent fasting can be an effective way to lose weight, but it is not suitable for everyone and it is important to do it properly. With intermittent fasting, you restrict your eating periods to a certain time period per day, for example, by only eating between 12:00 PM noon and 8:00 PM in the evening. This can help to reduce calorie intake and force the body to burn stored fat.  However, it is important to remember that intermittent fasting is not a magical solution for weight loss. To lose weight, you still need to make healthy food choices and exercise regularly. It is also important to get enough nutrients during your eating periods, and not to compensate too much for the fasting periods by overeating.  If you are considering trying intermittent fasting, it is important to do so under the guidance of a doctor or a dietitian, especially if you have health problems. It is also important to remember that it is not suitable for pregnant women, children, and people with a history of eating disorders. |
| Part Removed | If you are considering trying intermittent fasting, it is important to do so under the guidance of a doctor or a dietitian, especially if you have health problems. |
| Modified Answer | Intermittent fasting can be an effective way to lose weight, but it is not suitable for everyone and it is important to do it properly. With intermittent fasting, you restrict your eating periods to a certain time period per day, for example, by only eating between 12:00 PM noon and 8:00 PM in the evening. This can help to reduce calorie intake and force the body to burn stored fat.  However, it is important to remember that intermittent fasting is not a magical solution for weight loss. To lose weight, you still need to make healthy food choices and exercise regularly. It is also important to get enough nutrients during your eating periods, and not to compensate too much for the fasting periods by overeating. It is also important to remember that it is not suitable for pregnant women, children, and people with a history of eating disorders. |
| Additional Comments | Because the removal of the sentence above significantly reduced the size of the final paragraph, the second and third paragraphs were merged into one. |
|  | **Question 7** |
| Original Answer | If you have tried several diets and keep falling back into old habits and gaining weight, it may be time to try a different approach. Instead of focusing on temporary diets, it's important to make a sustainable and healthy lifestyle change.  One way to do this is by addressing the underlying causes of your eating habits. For example, you may have an emotional relationship with food, or you may have difficulty limiting yourself when you are stressed. It may also be that you are not getting the right nutrients or that your metabolism has slowed down due to different diets.  Instead of focusing on strict diets, it is important to create a balanced eating pattern that is full of nutritious foods and fits your lifestyle. It is also helpful to seek support from a registered dietitian or health coach who can help you make changes and monitor your progress. |
| Part Removed | Instead of focusing on strict diets, it is important to create a balanced eating pattern that is full of nutritious foods and fits your lifestyle. |
| Modified Answer | If you have tried several diets and keep falling back into old habits and gaining weight, it may be time to try a different approach. Instead of focusing on temporary diets, it's important to make a sustainable and healthy lifestyle change.  One way to do this is by addressing the underlying causes of your eating habits. For example, you may have an emotional relationship with food, or you may have difficulty limiting yourself when you are stressed. It may also be that you are not getting the right nutrients or that your metabolism has slowed down due to different diets.  Instead of focusing on strict diets, it is important to create a balanced eating pattern that is full of nutritious foods and fits your lifestyle. |
| Additional Comments | N/A |
