# Supplementary material for: Comparison of Answers between ChatGPT and Human Dieticians to Common Nutrition Questions
Source: J Nutr Metab. 2023 Nov 7;2023:5548684. doi: 10.1155/2023/5548684 (PMC10645493; doi:10.1155/2023/5548684)
Supplement: Supplementary Materials — Table S1: the two answers from ChatGPT that were modified before being sent for grading in their original form and after modification. Table S2: answers to each question from the dieticians and ChatGPT in Dutch and English. Table S3: the grade of each grading component and the average overall grade for the answer to every question for both ChatGPT and the dieticians. Table S4: summary statistics for the grades of the component scientific correctness. Table S5: summary statistics for the grades of the component actionability. Table S6: summary statistics for the grades of the component comprehensibility. Table S7: the p values of the permutation simulations of the test statistic with the mean and the median. [file 5548684.f1.zip › Table S2.docx]

Table S2: The questions and answers from dieticians and ChatGPT in Dutch (left half) and English (right)

|  | **Vraag** | **Antwoord** | **Question** | **Answer** |
| --- | --- | --- | --- | --- |
| *Q1* | *Kan ik intermittend fasting doen om sneller gewicht te verliezen?* | | *Can I use intermittent fasting to lose weight more quickly?* | |
|  | Diëtist | Gewicht verlies je door minder calorieën binnen te krijgen dan je verbruikt. Hierop zijn alle diëten in de media op gebaseerd. Intermittend fasting is een manier om minder calorieën binnen te krijgen, omdat je binnen een bepaald tijdsbestek maar mag eten. Voor iedereen is het persoonlijk wat goed werkt, dus als dit kader voor u werkt, kunt u dit zeker uitproberen. De belangrijke kanttekening is dat we in de tijd dat we mogen eten niet grotere porties dan normaal nemen, omdat anders de lagere calorie inname niet bereikt wordt. Verder is het altijd goed om op de eiwitinname te letten voor de verzadiging. | Dietician | You lose weight through consuming less calories than you use. This is the basis for all diets in the media. Intermittent fasting is a way to consume less calories because you restrict the time available in which you can eat. What works best varies between people, so if this approach works for you, then it’s definitely worth a try. The important footnote is that we do not eat larger portions than normal during the time in which we eat because otherwise calories won’t be reduced. Additionally, it’s always a good idea to consume protein to remain satiated. |
|  | ChatGPT | Intermittent fasting kan een effectieve manier zijn om gewicht te verliezen, maar het is niet voor iedereen geschikt en het is belangrijk om het op de juiste manier te doen. Bij intermittent fasting beperk je je eetperiodes tot een bepaalde tijdsperiode per dag, bijvoorbeeld door alleen te eten tussen 12:00 uur 's middags en 8:00 uur 's avonds. Dit kan helpen om de calorie-inname te verminderen en het lichaam te dwingen om opgeslagen vet te verbranden.  Het is echter belangrijk om te onthouden dat intermittent fasting geen magische oplossing is voor gewichtsverlies. Om af te vallen, moet je nog steeds gezonde voedingskeuzes maken en regelmatig bewegen. Het is ook belangrijk om voldoende voedingsstoffen binnen te krijgen tijdens je eetperiodes, en niet te veel te compenseren voor de vastenperiodes door overmatig te eten.  Als je overweegt om intermittent fasting te proberen, is het belangrijk om dit te doen onder begeleiding van een arts of een diëtist, vooral als je gezondheidsproblemen hebt. Het is ook belangrijk om te onthouden dat het niet geschikt is voor zwangere vrouwen, kinderen en mensen met een voorgeschiedenis van eetstoornissen. | ChatGPT | Intermittent fasting can be an effective way to lose weight, but it is not suitable for everyone and it is important to do it properly. With intermittent fasting, you restrict your eating periods to a certain time period per day, for example, by only eating between 12:00 PM noon and 8:00 PM in the evening. This can help to reduce calorie intake and force the body to burn stored fat.  However, it is important to remember that intermittent fasting is not a magical solution for weight loss. To lose weight, you still need to make healthy food choices and exercise regularly. It is also important to get enough nutrients during your eating periods, and not to compensate too much for the fasting periods by overeating. It is also important to remember that it is not suitable for pregnant women, children, and people with a history of eating disorders. |
| *Q2* | *Hoe kan ik smaakvol eten met minder zout?* | | *How can I eat tastily whilst using less salt?* | |
|  | Diëtist | Er steeds meer kruidenmixen beschikbaar zonder zout, zoals Verstegen met de blauwe dop of Jonnie Boer kruidenmixen. Het belangrijke om zout te minderen in uw eetpatroon is om minder te koken met pakjes en zakjes, minder voorbewerkte producten te nemen (denk aan gemarineerd vlees bijvoorbeeld), te letten op uw bouillonblokjes gebruik en uzelf de tijd te gunnen om te wennen aan de smaak. Het wennen aan minder zout duurt vaak rond de drie maanden. Ook kunt u kijken op de site van de nierstichting voor tips om met minder zout te koken. Zo staat er online een kruidenwijzer die aangeeft hoe u producten smakelijk kan klaarmaken en met welke kruiden. Ook kunt u uw eigen bouillon makkelijk zelf maken door aromatische groenten en kruiden te laten koken met water. Mocht u graag een vlees of vis bouillon willen kunt u de botten gebruiken om smaak te geven. | Dietician | There are more and more spice mixes available without salt, such as Verstegen with the blue cap or Jonnie Boer spice mix. Important for reducing salt intake in your diet is to use fewer packs, ready-made products (such as marinaded meat, for example), be careful with stock-cube use, and to give yourself enough time to adjust your taste to foods with less salt. The period of time required to adjust to lower salt is around three months. You can also look at the website of the Nierstichting for tips on how to cook with less salt. Here online, there is a herbal guide that indicates how products can be made tasty and which spices to use. You can also easily make stock yourself with aromatic vegetables and spices, and then cook with water. If you’d like a fish or meat bouillon, use the bones to provide flavour. |
|  | ChatGPT | Er zijn verschillende manieren om smaakvol te eten zonder al te veel zout toe te voegen. Hier zijn enkele tips:   1. Gebruik kruiden en specerijen: In plaats van zout, kun je kruiden en specerijen gebruiken om smaak toe te voegen aan je eten. Denk aan knoflook, uien, gember, chilipepers, basilicum, rozemarijn, tijm, enzovoort. 2. Experimenteer met azijn en citrus: Azijn en citrusvruchten, zoals citroen en limoen, kunnen je gerechten op smaak brengen zonder extra zout toe te voegen. 3. Maak zelf je sauzen en dressings: Veel kant-en-klare sauzen en dressings bevatten veel zout. Door zelf sauzen en dressings te maken, kun je de hoeveelheid zout beperken en nog steeds smaak toevoegen. 4. Kies voor verse ingrediënten: Vers voedsel heeft van nature meer smaak dan verwerkt voedsel. Probeer zoveel mogelijk verse ingrediënten te gebruiken in je maaltijden. 5. Probeer zoutvervangers: Er zijn verschillende zoutvervangers op de markt die natriumvrij of natriumarm zijn. Probeer er een paar uit en kijk welke je het lekkerst vindt.   Door deze tips te volgen, kun je smaakvol eten zonder al te veel zout toe te voegen. | ChatGPT | There are several ways to eat flavorful meals with less salt. Here are some tips:   1. Use herbs and spices: Instead of salt, you can use herbs and spices to add flavor to your food. Think garlic, onions, ginger, chili peppers, basil, rosemary, thyme, etc. 2. Experiment with vinegar and citrus: Vinegar and citrus fruits, such as lemon and lime, can add flavor to your dishes without adding extra salt. 3. Make your own sauces and dressings: Many pre-made sauces and dressings contain a lot of salt. By making your own sauces and dressings, you can limit the amount of salt and still add flavor. 4. Choose fresh ingredients: Fresh food has naturally more flavor than processed food. Try to use as many fresh ingredients as possible in your meals. 5. Try salt substitutes: There are several salt substitutes on the market that are sodium-free or low in sodium. Try a few and see which one you like best.   By following these tips, you can eat flavorful meals without adding too much salt. |
| *Q3* | *Zijn de suikers in fruit nu slecht om te nemen?* | | *Are the sugars in fruit bad to consume?* | |
|  | Diëtist | In veel producten zitten van nature suiker. Zo ook in fruit. Fruit biedt naast suiker ook vezels en vitaminen en is daardoor een voedingsmiddel wat hoort in ons dagelijks eetpatroon. Als u wilt minderen in suiker, is het belangrijker om geen toegevoegde suiker tot u te nemen. Dit houdt naast tafelsuiker ook kokosbloesemsuiker of honing in. Dit ziet het lichaam allemaal als puur suiker en heeft geen andere voedingswaarden die van belang zijn voor het lichaam.  Verder is het beter om fruit te eten dan te drinken, daarmee bedoelen we dat we liever een stuk fruit moeten eten dan vruchtensap drinken. Dit omdat we een deel van de vezels zullen missen, die juist goed zijn voor uw gezondheid. | Dietician | Many products contain natural sugars. This is also true for fruit. Apart from sugar, fruit also provides fibers and vitamins and therefore belongs in our daily eating pattern. If you would like to reduce your sugar, it is more important to consume no added sugar. This holds not only for table sugar but also coconut blossom sugar and honey. The body sees this all as pure sugar, and without any other important nutritional value. It is also better to eat rather than drink fruit, and what we mean by this is that it is better to eat a piece of fruit than to drink fruit juice. This is because we miss part of the fiber that is also good for your health. |
|  | ChatGPT | De suikers in fruit zijn van nature aanwezig en verschillen van de toegevoegde suikers in verwerkte voedingsmiddelen. Het eten van fruit als onderdeel van een gezond dieet is over het algemeen niet slecht. In feite bevatten veel soorten fruit naast suikers ook vezels, vitamines, mineralen en andere voedingsstoffen die belangrijk zijn voor een gezond lichaam.  Hoewel fruit dus gezond is, is het belangrijk om te onthouden dat sommige soorten fruit meer suikers bevatten dan andere. Zo bevatten bijvoorbeeld bananen en druiven meer suikers dan bessen en appels. Mensen met diabetes moeten daarom extra voorzichtig zijn met hun fruitinname en overleggen met een diëtist of arts over welke soorten fruit het beste voor hen zijn.  Als je geen gezondheidsproblemen hebt, kan het eten van fruit als onderdeel van een gevarieerd dieet gunstig zijn voor je gezondheid. Het is echter wel belangrijk om de inname van toegevoegde suikers uit andere voedingsmiddelen te beperken. | ChatGPT | The sugars in fruit are naturally occurring and differ from added sugars in processed foods. Eating fruit as part of a healthy diet is generally not bad. In fact, many types of fruit contain not only sugars but also fiber, vitamins, minerals, and other nutrients that are important for a healthy body.  While fruit is healthy, it is important to remember that some types of fruit contain more sugars than others. For example, bananas and grapes contain more sugar than berries and apples. People with diabetes should therefore be extra careful with their fruit intake and consult with a dietitian or doctor about which types of fruit are best for them.  If you don't have any health problems, eating fruit as part of a varied diet can be beneficial to your health. However, it is important to limit the intake of added sugars from other foods. |
| *Q4* | *Zijn koolhydraten slecht?* | | *Are carbohydrates bad?* | |
|  | Diëtist | Eén specifiek product of productgroep maakt een keuze niet “slecht”. Het is belangrijk om te kijken naar het gehele plaatje. Koolhydraten zijn een energieleverancier voor het lijf en je vindt ze in veel producten. Denk bijvoorbeeld aan graanproducten zoals brood, pasta, rijst etc. Ook in zuivel (melksuiker), fruit (fruitsuiker) en peulvruchten vind je koolhydraten. Een gehele productgroep zoals koolhydraten weglaten raad ik af: het is a) vaak moeilijk vol te houden en b) je hebt koolhydraten nodig voor de energie, vezels, vitaminen en mineralen. Kies vooral voor de langzame koolhydraten, oftewel de volkoren producten en kijk of je ze kan combineren met eiwitten en vetten. En het allerbelangrijkste: blijf variëren binnen je eetpatroon en ontdek wat jij lekker vindt en wat jouw een verzadigd en voldaan gevoel geeft. | Dietician | One specific product or product group doesn’t make a choice “bad”. It is important to look at the whole picture. Carbohydrates are an energy source for life and you find them in so many products. Think, for example, of grain products such as bread, pasta, rice, etc. Also in dairy (milk sugar), fruit (fruit sugar), and legumes you also find carbohydrates. I advise against excluding a whole product group such as carbohydrates: it is a) often difficult to maintain and b) you need carbohydrates for energy, fiber, vitamins and minerals. Opt for slow carbohydrates, in other words whole grain products, and see if you can combine them with protein and fat. Most importantly: keep variety in your eating pattern and discover what you enjoy and leaves you feeling full and satisfied. |
|  | ChatGPT | Koolhydraten zijn een belangrijke bron van energie voor ons lichaam en zijn niet per definitie slecht. Er zijn verschillende soorten koolhydraten, zoals enkelvoudige en complexe koolhydraten. Enkelvoudige koolhydraten worden snel afgebroken en kunnen zorgen voor een snelle stijging van de bloedsuikerspiegel, terwijl complexe koolhydraten langzamer worden afgebroken en zorgen voor een geleidelijke stijging van de bloedsuikerspiegel.  Het is belangrijk om te onthouden dat niet alle koolhydraten gelijk zijn. Koolhydraten uit volkorenproducten, groenten, fruit en peulvruchten zijn gezonde bronnen van koolhydraten en bevatten ook vezels, vitamines en mineralen die belangrijk zijn voor onze gezondheid. Aan de andere kant bevatten bewerkte voedingsmiddelen zoals snoep, gebak en wit brood vaak veel toegevoegde suikers en witte bloem, waardoor ze minder voedzaam zijn en kunnen bijdragen aan gewichtstoename en andere gezondheidsproblemen.  Het is daarom belangrijk om koolhydraten in balans te eten en te kiezen voor gezonde koolhydraatbronnen als onderdeel van een uitgebalanceerd dieet. | ChatGPT | Carbohydrates are an important source of energy for our bodies and are not necessarily bad. There are different types of carbohydrates, such as simple and complex carbohydrates. Simple carbohydrates are quickly broken down and can cause a rapid increase in blood sugar levels, while complex carbohydrates are broken down more slowly and cause a gradual increase in blood sugar levels.  It is important to remember that not all carbohydrates are created equal. Carbohydrates from whole grains, vegetables, fruit, and legumes are healthy sources of carbohydrates and also contain fiber, vitamins, and minerals that are important for our health. On the other hand, processed foods such as candy, pastries, and white bread often contain a lot of added sugars and white flour, making them less nutritious and can contribute to weight gain and other health problems.  Therefore, it is important to eat carbohydrates in balance and choose healthy carbohydrate sources as part of a balanced diet. |
| *Q5* | *Kan ik suiker beter vervangen door honing? Of zoetstof?* | | *Is it better to swap sugar for honey? Or sweeteners?* | |
|  | Diëtist | Ik denk dat het goed is om het gebruik van toegevoegd suiker zo veel mogelijk te matigen. Honing valt hier ook onder. Je lijf herkent namelijk niet of je honing of suiker in bijvoorbeeld je thee doet. Suikerrijke producten geven je lichaam snel energie, maar ook voor korte duur. Dit kan schommelingen veroorzaken in je bloedsuikerspiegel; wanneer je suikers eet/drinkt gaat de suikerspiegel omhoog (je voelt je energieker), na een tijdje zakt deze weer omlaag (je voelt een ‘dipje’). Zoetstoffen zijn chemische zoete smaakmakers. In tegenstelling tot gewone tafelsuiker of honing geven de meeste zoetstoffen geen stijging van je suikerspiegel en leveren ze geen of nauwelijks calorieën. Je vindt ze veelal in light en zero producten. Het gebruik van producten met zoetstoffen raad ik af, of beperk het in ieder geval zoveel mogelijk. Het is nog niet duidelijk wat zoetstoffen op de lange termijn met onze gezondheid doet. Dus: wees matig met toegevoegd suikers en zoetstoffen, af en toe een zoete lekkernij is prima, maar kies bewust. | Dietician | I think it’s good to limit the use of added sugar as much as possible. That includes honey. Your body doesn’t know whether you’ve put honey or sugar in your tea. Sugar-rich products give your body quick energy but for short periods. This can cause fluctuations in your blood sugar; when you eat/drink sugars, sugar levels rise (you feel more energetic), and after a period of time they fall again (you feel a “dip”). Sweeteners are chemical sweet seasoners. In contrast to normal table sugar or honey, most sweeteners do not increase blood sugar and provide hardly any calories. You find them in many light or diet products. I advise against the use of sweetened products or at least limit them as much as possible. It is not yet clear how sweeteners affect our health in the long term. So, moderate added sugars and sweeteners, a sweet treat here and there is fine, but choose mindfully. |
|  | ChatGPT | Het is begrijpelijk dat mensen op zoek zijn naar alternatieven voor suiker om hun inname van geraffineerde suikers te verminderen. Honing wordt vaak gezien als een gezonder alternatief voor suiker omdat het natuurlijk is en antioxidanten, mineralen en vitamines bevat. Hoewel honing wel gezonde voedingsstoffen bevat, bevat het ook veel fructose en glucose, wat hetzelfde effect op de bloedsuikerspiegel kan hebben als suiker. Bovendien bevat honing ook veel calorieën, dus het is belangrijk om het met mate te consumeren.  Zoetstoffen zoals aspartaam, saccharine en sucralose worden vaak gebruikt als vervangers voor suiker omdat ze weinig calorieën bevatten. Hoewel ze veilig zijn bevonden door voedselveiligheidsinstanties, is er enige bezorgdheid over de langetermijneffecten van het gebruik van deze stoffen. Sommige studies suggereren dat het gebruik van zoetstoffen kan leiden tot gewichtstoename en andere gezondheidsproblemen.  Het is daarom belangrijk om te onthouden dat hoewel vervangers voor suiker een goede manier kunnen zijn om de inname van geraffineerde suikers te verminderen, het belangrijk is om in balans te blijven en te kiezen voor natuurlijke bronnen van zoetheid, zoals fruit. | ChatGPT | It's understandable that people are looking for alternatives to sugar to reduce their intake of refined sugars. Honey is often seen as a healthier alternative to sugar because it is natural and contains antioxidants, minerals, and vitamins. While honey does contain healthy nutrients, it also contains a lot of fructose and glucose, which can have the same effect on blood sugar levels as sugar. Additionally, honey also contains a lot of calories, so it's important to consume it in moderation.  Artificial sweeteners such as aspartame, saccharin, and sucralose are often used as substitutes for sugar because they contain few calories. While they have been deemed safe by food safety agencies, there is some concern about the long-term effects of using these substances. Some studies suggest that the use of artificial sweeteners can lead to weight gain and other health problems.  It's important to remember that while sugar substitutes can be a good way to reduce the intake of refined sugars, it's important to stay balanced and choose natural sources of sweetness, such as fruit. |
| *Q6* | *Wat is een gezond tussendoortje?* | | *What is a healthy snack?* | |
|  | Diëtist | Probeer zoveel mogelijk producten te gebruiken uit de categorie ‘basisvoedingsmiddelen’, ofwel producten die in de schijf van vijf staan. Denk bijvoorbeeld aan snackgroenten, fruit, ongezouten nootjes, volkorencracker met beleg. Vermijd producten die heel erg bewerkt zijn (‘ultra processed foods’), deze producten hebben vaak een slechtere voedingswaarde en geven een slecht verzadigd gevoel. Het zijn geen verboden producten, maar kies toch liever voor zoveel mogelijk verse en onbewerkte producten. | Dietician | Aim as much as possible to go for products from the “staple foods” category or products in the disk of five. Think for example of vegetable snacks, fruit, unsalted nuts, whole grain crackers with toppings. Avoid ultra-processed foods, these products often have worse nutrition value and poor satiation. They’re not to be prohibited, but rather opt instead for fresh and unprocessed products as much as possible. |
|  | ChatGPT | Een gezond tussendoortje kan bestaan uit een combinatie van eiwitten, vezels en gezonde vetten, die allemaal helpen om je langer verzadigd te houden en je bloedsuikerspiegel stabiel te houden.  Enkele voorbeelden van gezonde tussendoortjes zijn:   - Een stuk fruit met een handjevol noten of zaden - Groentesticks met hummus, guacamole of een andere gezonde dipsaus - Griekse yoghurt met bessen en een beetje honing - Een hardgekookt ei met een stuk volkoren toast - Een handjevol gedroogde vruchten en ongezouten noten - Een volkoren cracker met plakjes avocado en tomaat   Het is ook belangrijk om te onthouden dat portiegrootte een belangrijke factor is bij het kiezen van gezonde tussendoortjes. Het is gemakkelijk om te veel te eten, zelfs als het gezond voedsel is. Een portie van ongeveer 150 tot 200 calorieën is meestal voldoende voor een tussendoortje. | ChatGPT | A healthy snack can consist of a combination of protein, fiber, and healthy fats, all of which help keep you feeling full longer and maintain stable blood sugar levels.  Some examples of healthy snacks include:   - A piece of fruit with a handful of nuts or seeds - Veggie sticks with hummus, guacamole, or another healthy dip - Greek yogurt with berries and a bit of honey - A hard-boiled egg with a slice of whole wheat toast - A handful of dried fruits and unsalted nuts - A whole grain cracker with avocado and tomato slices   It's also important to remember that portion size is a key factor in choosing healthy snacks. It's easy to overeat, even if it's healthy food. A serving size of about 150 to 200 calories is usually sufficient for a snack. |
| *Q7* | *Ik heb al veel diëten geprobeerd de afgelopen jaren. Ik verlies dan steeds gewicht, maar kom ook weer aan.* | | *I’ve tried many diets in the last few years. I lose weight, but then it comes back.* | |
|  | Diëtist | Ik leg uit waarom diëten niet werken. Het is een tijdelijke oplossing wat resultaat kan hebben. Als je vervolgens stopt met het dieet en doet wat je altijd deed, dan kom je weer aan in gewicht. Vaak is dit meer dan het eerdere startgewicht. Ik leg mensen uit dat ik niet werk met standaard dieten, maar een schema/adviezen maak op basis van hun huidige eetpatroon. Hierdoor kan er met relatief kleine veranderingen, gezorgd worden voor minder energie inname (mager ipv vol, 30+ ipv 48+ kaas, etc.). Ik leg uit dat het een verandering van leefstijl is en geen dieet. Er is dus ook zeker ruimte voor chocola/koekjes oid. Belangrijk om na te gaan waarom je dit wil eten (is het uit emotie of omdat je het lekker vindt en van gaat genieten). Eet het vervolgens ook bewust en niet voor de tv een zak leeg. | Dietician | I will explain why diets don’t work. It is a temporary solution that can lead to results. If you then stop the diet and do what you’ve always done, then the weight comes right back. Often, this is more than the start weight. I explain to people that I don’t work with standard diets, but make a plan/give advice based on their current eating pattern. This allows for a lower energy intake with relatively small changes (thin instead of full, 30+ instead of 48+ cheese, etc.). I explain that a change in lifestyle is not a diet. There is also, therefore, definitely room for chocolate/cookies. It is important to know why you want to eat this (is it out of emotions or because you like the taste). Eat it mindfully and not from the bag in front of the TV. |
|  | ChatGPT | Als je verschillende diëten hebt geprobeerd en steeds weer terugvalt in oude gewoonten en gewicht terugkrijgt, kan het tijd zijn om een andere aanpak te proberen. In plaats van je te concentreren op tijdelijke diëten, is het belangrijk om een duurzame en gezonde levensstijlverandering te maken.  Een manier om dit te doen is door het aanpakken van de onderliggende oorzaken van jouw eetgewoonten. Misschien heb je bijvoorbeeld een emotionele relatie met voedsel, of heb je moeite om jezelf te beperken als je gestrest bent. Het kan ook zijn dat je niet de juiste voedingsstoffen binnenkrijgt of dat je metabolisme langzamer is geworden door verschillende diëten.  In plaats van te focussen op streng diëten, is het belangrijk om een gebalanceerd eetpatroon te creëren dat vol zit met voedzame voedingsmiddelen en dat past bij jouw levensstijl. Het is ook nuttig om ondersteuning te zoeken bij een diëtist of een gezondheidscoach die jou kan helpen om veranderingen aan te brengen en jouw vooruitgang te monitoren. | ChatGPT | If you have tried several diets and keep falling back into old habits and gaining weight, it may be time to try a different approach. Instead of focusing on temporary diets, it's important to make a sustainable and healthy lifestyle change.  One way to do this is by addressing the underlying causes of your eating habits. For example, you may have an emotional relationship with food, or you may have difficulty limiting yourself when you are stressed. It may also be that you are not getting the right nutrients or that your metabolism has slowed down due to different diets.  Instead of focusing on strict diets, it is important to create a balanced eating pattern that is full of nutritious foods and fits your lifestyle. |
| *Q8* | *Of vitamine/mineralen suppletie nodig is* | | *Is vitamin/mineral supplementation necessary?* | |
|  | Diëtist | Mocht je bij een specifieke doelgroep horen, dan kan het voor bepaalde micronutriënten wel het geval zijn. Mocht je gevarieerd en voldoende eten, is het over het algemeen niet nodig. In de winter kan je wel baat hebben bij vit D (zeker als je geen of weinig smeervet gebruikt en bakt in olijfolie). Je kunt een laag gedoseerde multivitamine slikken (tot max 150% van de ADH). Hoog gedoseerd raad ik nooit aan, zonde van je geld en nergens voor nodig. Van sommige micronutriënten kan het zelfs schadelijk zijn om er veel te veel van binnen te krijgen. | Dietician | If you belong to a specific target group, then it may be the case for certain micronutrients. If you have a varied and satisfactory diet, then it is generally not necessary. In the winter, you might benefit from vitamin D (especially if you use little to no grease fat and cook with olive oil). You can use a low dose multivitamin (up to a maximum of 150% of the RDA). I never recommend high doses, which are a waste of money and never necessary. For some micronutrients, it is even harmful to consume too much. |
|  | ChatGPT | Of je extra vitamines of supplementen nodig hebt, hangt af van verschillende factoren, waaronder je dieet, levensstijl en gezondheidstoestand. Over het algemeen kunnen de meeste mensen alle benodigde voedingsstoffen binnenkrijgen via een gezond en gevarieerd dieet.  Als je echter een restrictief dieet volgt, zoals een veganistisch of vegetarisch dieet, kan het zijn dat je bepaalde voedingsstoffen mist en dat het aanbevolen is om supplementen te nemen. Ook bij bepaalde gezondheidstoestanden kan een arts of diëtist aanbevelen om bepaalde supplementen te nemen.  Het is belangrijk om op te merken dat het nemen van te veel vitamines of supplementen ook schadelijk kan zijn voor je gezondheid. Daarom is het belangrijk om altijd de aanbevolen doseringen te volgen en professioneel advies in te winnen voordat je supplementen neemt. | ChatGPT | Whether you need extra vitamins or supplements depends on several factors, including your diet, lifestyle, and health status. Generally, most people can get all the necessary nutrients through a healthy and varied diet.  However, if you follow a restrictive diet such as a vegan or vegetarian diet, you may be missing certain nutrients and it may be recommended to take supplements. Also, in certain health conditions, a doctor or dietitian may recommend taking certain supplements.  It is important to note that taking too much vitamins or supplements can also be harmful to your health. Therefore, it is important to always follow recommended dosages before taking supplements. |
