# Supplementary material for: Comparison of Answers between ChatGPT and Human Dieticians to Common Nutrition Questions
Source: J Nutr Metab. 2023 Nov 7;2023:5548684. doi: 10.1155/2023/5548684 (PMC10645493; doi:10.1155/2023/5548684)
Supplement: Supplementary Materials — Table S1: the two answers from ChatGPT that were modified before being sent for grading in their original form and after modification. Table S2: answers to each question from the dieticians and ChatGPT in Dutch and English. Table S3: the grade of each grading component and the average overall grade for the answer to every question for both ChatGPT and the dieticians. Table S4: summary statistics for the grades of the component scientific correctness. Table S5: summary statistics for the grades of the component actionability. Table S6: summary statistics for the grades of the component comprehensibility. Table S7: the p values of the permutation simulations of the test statistic with the mean and the median. [file 5548684.f1.zip › Table S3.docx]

| Table S3 The grade of each grading component and the average overall grade for the answer to every question for both ChatGPT and the dieticians. | | | | | | | | | | | | | | | | | | | | | | | | | | | | | | | | | | | | | | | | | | | | | | | | | |  |
| --- | --- | --- | --- | --- | --- | --- | --- | --- | --- | --- | --- | --- | --- | --- | --- | --- | --- | --- | --- | --- | --- | --- | --- | --- | --- | --- | --- | --- | --- | --- | --- | --- | --- | --- | --- | --- | --- | --- | --- | --- | --- | --- | --- | --- | --- | --- | --- | --- | --- | --- |
|  |  |  |  |  |  |  |  |  |  |  |  |  |  |  |  |  |  |  |  |  |  |  |  |  |  |  |  |  |  |  |  |  |  |  |  |  |  |  |  |  |  |  |  |  |  |  |  |  |  |  |
|  |  |  |  |  |  |  |  |  |  |  |  |  |  |  |  |  |  |  |  |  |  |  |  |  |  |  |  |  |  |  |  |  |  |  |  |  |  |  |  |  |  |  |  |  |  |  |  |  |  |  |
|  |  |  |  |  |  |  |  |  |  |  |  |  |  |  |  |  |  |  |  |  |  |  |  |  |  |  |  |  |  |  |  |  |  |  |  |  |  |  |  |  |  |  |  |  |  |  |  |  |  |  |
|  | | DIETICIAN | | | CHATGPT | | | DIETICIAN | | | CHATGPT | | | DIETICIAN | | | CHATGPT | | | DIETICIAN | | | CHATGPT | | | DIETICIAN | | | CHATGPT | | | DIETICIAN | | | CHATGPT | | | DIETICIAN | | | CHATGPT | | | DIETICIAN | | | CHATGPT | | |  |
|  |  | 1 | | | 1 | | | 2 | | | 2 | | | 3 | | | 3 | | | 4 | | | 4 | | | 5 | | | 5 | | | 6 | | | 6 | | | 7 | | | 7 | | | 8 | | | 8 | | |  |
|  |  | SC | C | A | SC | C | A | SC | C | A | SC | C | A | SC | C | A | SC | C | A | SC | C | A | SC | C | A | SC | C | A | SC | C | A | SC | C | A | SC | C | A | SC | C | A | SC | C | A | SC | C | A | SC | C | A |  |
| GRADES | DIETICIANS | 6 | 7 | 5 | 7 | 8 | 5 | 6 | 8 | 8 | 5 | 8 | 8 | 7 | 7 | 6 | 8 | 8 | 6 | 7 | 7 | 7 | 9 | 8 | 7 | 8 | 8 | 8 | 6 | 8 | 7 | 7 | 6 | 5 | 9 | 9 | 9 | 5 | 5 | 3 | 9 | 8 | 6 | 5 | 8 | 8 | 9 | 9 | 8 |  |
|  |  | 8 | 6 | 6 | 7 | 9 | 8 | 7 | 5 | 6 | 9 | 10 | 9 | 8 | 6 | 8 | 9 | 9 | 9 | 8 | 6 | 6 | 8 | 8 | 7 | 4 | 7 | 6 | 5 | 8 | 8 | 5 | 5 | 5 | 7 | 9 | 9 | 7 | 8 | 6 | 7 | 9 | 8 | 6 | 5 | 5 | 9 | 9 | 9 |  |
|  |  | 8 | 7 | 4 | 10 | 10 | 8 | 9 | 9 | 10 | 8 | 10 | 9 | 10 | 10 | 10 | 10 | 8 | 5 | 9 | 5 | 5 | 10 | 8 | 8 | 8 | 7 | 5 | 8 | 8 | 10 | 9 | 7 | 5 | 8 | 9 | 9 | 10 | 8 | 7 | 10 | 8 | 7 | 9 | 3 | 3 | 10 | 9 | 10 |  |
|  |  | 9 | 7 | 8 | 10 | 8 | 7 | 9 | 6 | 7 | 8 | 9 | 9 | 9 | 9 | 9 | 8 | 7 | 8 | 10 | 10 | 9 | 8 | 8 | 7 | 9 | 7 | 7 | 9 | 8 | 9 | 6 | 7 | 7 | 10 | 10 | 10 | 7 | 6 | 6 | 7 | 9 | 8 | 7 | 7 | 7 | 8 | 8 | 7 |  |
|  |  | 6 | 9 | 5 | 7 | 6 | 7 | 8 | 8 | 8 | 8 | 8 | 9 | 9 | 7 | 8 | 7 | 8 | 6 | 9 | 8 | 9 | 10 | 9 | 10 | 5 | 8 | 3 | 6 | 5 | 5 | 9 | 7 | 6 | 7 | 6 | 5 | 9 | 8 | 8 | 10 | 10 | 9 | 4 | 6 | 3 | 9 | 9 | 9 |  |
|  |  | 5 | 4 | 5 | 7 | 7 | 7 | 4 | 6 | 5 | 8 | 8 | 8 | 8 | 7 | 7 | 9 | 8 | 8 | 5 | 5 | 6 | 8 | 8 | 8 | 5 | 7 | 7 | 9 | 6 | 5 | 9 | 8 | 7 | 8 | 8 | 8 | 5 | 7 | 6 | 9 | 8 | 8 | 8 | 7 | 7 | 9 | 8 | 8 |  |
|  |  | 8 | 6 | 7 | 9 | 9 | 9 | 9 | 8 | 9 | 8 | 9 | 9 | 8 | 7 | 7 | 7 | 8 | 7 | 8 | 8 | 8 | 6 | 6 | 7 | 5 | 7 | 6 | 6 | 7 | 6 | 7 | 8 | 7 | 8 | 8 | 7 | 7 | 8 | 8 | 7 | 7 | 7 | 7 | 7 | 7 | 8 | 8 | 8 |  |
|  |  | 8 | 8 | 6 | 10 | 10 | 8 | 10 | 10 | 10 | 6 | 9 | 8 | 4 | 6 | 7 | 5 | 8 | 8 | 10 | 8 | 10 | 10 | 6 | 6 | 10 | 6 | 6 | 10 | 6 | 6 | 10 | 10 | 10 | 6 | 8 | 8 | 10 | 10 | 10 | 8 | 6 | 5 | 10 | 10 | 10 | 10 | 8 | 9 |  |
|  |  | 5 | 6 | 5 | 8 | 10 | 8 | 10 | 10 | 10 | 8 | 8 | 7 | 9 | 8 | 7 | 7 | 8 | 5 | 5 | 5 | 5 | 10 | 10 | 10 | 8 | 10 | 8 | 10 | 8 | 8 | 5 | 7 | 5 | 10 | 10 | 10 | 6 | 6 | 5 | 10 | 10 | 10 | 5 | 6 | 5 | 7 | 7 | 7 |  |
|  |  | 7 | 6 | 6 | 7 | 8 | 8 | 8 | 8 | 8 | 7 | 8 | 7 | 7 | 7 | 6 | 6 | 7 | 6 | 9 | 8 | 7 | 9 | 8 | 8 | 8 | 8 | 8 | 7 | 6 | 6 | 7 | 8 | 7 | 9 | 8 | 9 | 7 | 6 | 6 | 8 | 8 | 7 | 6 | 6 | 6 | 8 | 8 | 7 |  |
|  |  | 3 | 7 | 6 | 5 | 6 | 4 | 6 | 6 | 4 | 5 | 9 | 8 | 6 | 7 | 8 | 8 | 6 | 5 | 6 | 7 | 7 | 8 | 6 | 7 | 5 | 7 | 4 | 7 | 5 | 4 | 6 | 8 | 8 | 6 | 8 | 9 | 5 | 8 | 8 | 7 | 7 | 6 | 6 | 6 | 4 | 8 | 6 | 4 |  |
|  |  | 8 | 8 | 7 | 6 | 7 | 5 | 6 | 4 | 5 | 9 | 9 | 9 | 7 | 5 | 6 | 7 | 8 | 7 | 7 | 6 | 5 | 8 | 9 | 8 | 7 | 5 | 7 | 6 | 9 | 8 | 7 | 4 | 6 | 9 | 9 | 9 | 7 | 3 | 3 | 8 | 8 | 7 | 7 | 6 | 7 | 9 | 7 | 8 |  |
|  |  | 5 | 6 | 6 | 6 | 8 | 7 | 6 | 6 | 6 | 8 | 8 | 8 | 6 | 6 | 5 | 6 | 5 | 5 | 8 | 6 | 6 | 8 | 8 | 8 | 6 | 7 | 8 | 8 | 7 | 6 | 7 | 6 | 6 | 6 | 8 | 7 | 5 | 5 | 5 | 7 | 8 | 8 | 5 | 5 | 5 | 6 | 5 | 6 |  |
|  |  | 0 | 6 | 0 | 0 | 6 | 0 | 8 | 5 | 6 | 8 | 7 | 7 | 7 | 5 | 5 | 6 | 6 | 6 | 9 | 8 | 8 | 8 | 6 | 6 | 4 | 4 | 4 | 8 | 8 | 7 | 8 | 5 | 5 | 7 | 7 | 7 | 5 | 5 | 5 | 7 | 7 | 7 | 6 | 6 | 6 | 8 | 8 | 7 |  |
|  |  | 6 | 3 | 3 | 8 | 8 | 6 | 5 | 8 | 8 | 8 | 9 | 10 | 6 | 7 | 7 | 9 | 9 | 9 | 7 | 5 | 3 | 9 | 9 | 8 | 6 | 6 | 6 | 5 | 8 | 6 | 7 | 4 | 4 | 8 | 7 | 7 | 6 | 5 | 5 | 9 | 8 | 9 | 2 | 4 | 4 | 7 | 7 | 7 |  |
|  |  | 6 | 6 | 6 | 6 | 5 | 6 | 6 | 6 | 6 | 6 | 7 | 6 | 6 | 5 | 5 | 6 | 6 | 6 | 7 | 6 | 6 | 6 | 6 | 5 | 5 | 5 | 5 | 6 | 5 | 5 | 6 | 5 | 5 | 5 | 6 | 6 | 6 | 6 | 6 | 7 | 6 | 6 | 6 | 5 | 5 | 7 | 6 | 6 |  |
|  |  | 9 | 9 | 8 | 9 | 9 | 9 | 10 | 10 | 10 | 9 | 9 | 10 | 10 | 10 | 10 | 10 | 10 | 9 | 10 | 10 | 10 | 10 | 10 | 10 | 10 | 10 | 8 | 10 | 10 | 9 | 10 | 10 | 7 | 10 | 10 | 10 | 10 | 10 | 10 | 10 | 10 | 8 | 10 | 8 | 7 | 10 | 10 | 9 |  |
|  |  | 7 | 10 | 7 | 7 | 10 | 8 | 9 | 7 | 7 | 8 | 10 | 9 | 9 | 10 | 8 | 8 | 10 | 8 | 10 | 10 | 10 | 10 | 10 | 9 | 9 | 10 | 9 | 8 | 10 | 9 | 7 | 8 | 6 | 10 | 10 | 10 | 7 | 8 | 8 | 8 | 9 | 8 | 9 | 9 | 7 | 10 | 10 | 8 |  |
|  | EXPERTS | 6 | 8 | 7 | 7 | 9 | 7 | 6.5 | 6 | 6 | 8 | 7 | 7 | 8 | 9 | 8 | 6 | 9 | 7 | 8 | 8 | 7 | 8 | 9 | 9 | 3 | 8 | 8 | 4 | 8 | 8 | 7 | 6 | 7 | 9 | 10 | 10 | 9 | 8 | 8 | 8 | 8 | 7 | 8 | 10 | 8 | 10 | 10 | 8 |  |
|  |  | 6 | 6 | 5 | 8 | 9 | 8 | 9 | 7 | 7 | 7 | 8 | 8 | 7 | 6 | 9 | 9 | 9 | 8 | 6 | 7 | 9 | 9 | 9 | 8 | 6 | 7 | 9 | 10 | 9 | 10 | 10 | 6 | 7 | 8 | 10 | 10 | 8 | 8 | 3 | 8 | 10 | 7 | 5 | 6 | 6 | 9 | 6.5 | 7 |  |
|  |  | 8 | 7 | 5 | 6 | 9 | 7 | 7 | 7 | 5 | 7 | 8 | 7 | 8 | 7 | 7 | 9 | 8 | 8 | 9 | 8 | 8 | 8 | 7 | 8 | 8 | 7 | 7 | 9 | 8 | 8 | 6 | 6 | 6 | 7 | 8 | 6.5 | 6 | 7 | 4 | 5 | 7 | 4 | 5 | 5 | 4 | 7 | 7 | 6 |  |
|  |  | 7 | 8 | 6 | 8 | 7 | 5 | 7 | 6.5 | 8 | 6.5 | 8 | 8 | 7.5 | 6.5 | 5 | 6.5 | 6 | 5 | 7 | 6.5 | 6 | 7.5 | 6 | 6 | 4 | 6 | 5 | 4 | 7 | 6.5 | 7 | 7 | 7 | 5.5 | 8 | 8 | 5.5 | 6 | 4 | 8 | 8 | 7 | 7 | 6 | 7 | 8 | 7 | 6 |  |
|  |  | 8 | 6 | 5 | 8 | 9 | 9 | 8 | 4 | 5 | 8 | 6 | 6 | 10 | 8 | 7 | 8 | 7 | 7 | 7 | 7 | 6 | 9 | 8 | 9 | 9 | 9 | 9 | 8 | 6 | 6 | 7 | 5 | 5 | 8 | 7 | 8 | 7 | 8 | 6 | 4 | 8 | 8 | 6.5 | 5.5 | 55 | 8 | 7 | 7 |  |
|  |  | 8 | 8 | 7 | 9 | 9 | 8 | 8 | 7 | 6 | 8 | 8 | 8 | 8 | 8 | 8 | 6 | 6 | 6 | 7 | 7 | 7 | 7 | 8 | 8 | 3 | 8 | 9 | 3 | 6 | 7 | 9 | 7 | 7 | 9 | 9 | 9 | 7 | 7 | 7 | 9 | 9 | 9 | 6 | 6 | 6 | 8 | 7 | 5 |  |
|  |  | 10 | 10 | 8 | 10 | 5 | 7 | 8 | 6 | 7 | 7 | 8 | 4 | 7 | 4 | 5 | 4 | 6 | 6 | 5 | 7 | 7 | 7 | 4 | 7 | 8 | 6 | 6 | 8 | 4 | 7 | 9 | 8 | 8 | 7 | 6 | 9 | 9 | 8 | 7 | 7 | 6 | 6 | 6 | 7 | 8 | 8 | 4 | 5 |  |
|  |  | 9 | 8 | 8 | 9 | 9 | 9 | 9 | 7 | 8 | 8 | 8 | 8 | 7 | 10 | 10 | 8 | 10 | 10 | 5 | 5 | 10 | 7 | 10 | 10 | 6 | 7 | 10 | 7 | 7 | 10 | 6 | 6 | 8 | 10 | 8 | 10 | 10 | 10 | 10 | 10 | 6 | 6 | 6 | 6 | 6 | 6 | 7 | 6 |  |
|  |  | 9 | 6 | 7 | 7 | 7 | 5 | 9 | 7 | 6 | 6 | 8 | 8 | 8 | 8 | 9 | 9 | 5 | 5 | 8 | 7 | 7 | 9 | 6 | 5 | 7 | 8 | 8 | 9 | 5 | 6 | 7 | 8 | 6 | 4 | 8 | 8 | 8 | 7 | 8 | 9 | 9 | 9 | 6 | 5 | 7 | 9 | 8 | 5 |  |
|  | TOTAL | 6.85 | 6.96 | 5.85 | 7.44 | 8.04 | 6.85 | 7.69 | 6.94 | 7.07 | 7.46 | 8.3 | 7.93 | 7.65 | 7.24 | 7.3 | 7.46 | 7.59 | 6.85 | 7.63 | 7.06 | 7.19 | 8.39 | 7.78 | 7.74 | 6.52 | 7.22 | 6.89 | 7.26 | 7.11 | 7.13 | 7.41 | 6.74 | 6.37 | 7.8 | 8.3 | 8.43 | 7.17 | 7.07 | 6.37 | 8 | 8.04 | 7.3 | 6.43 | 6.31 | 7.89 | 8.33 | 7.61 | 7.11 |  |
|  |  | 6.555555556 | | | 7.444444444 | | | 7.234567901 | | | 7.895061728 | | | 7.395061728 | | | 7.302469136 | | | 7.290123457 | | | 7.969135802 | | | 6.87654321 | | | 7.166666667 | | | 6.839506173 | | | 8.172839506 | | | 6.87037037 | | | 7.777777778 | | | 6.87654321 | | | 7.685185185 | | |  |
