# Supplementary material for: Comparison of Answers between ChatGPT and Human Dieticians to Common Nutrition Questions
Source: J Nutr Metab. 2023 Nov 7;2023:5548684. doi: 10.1155/2023/5548684 (PMC10645493; doi:10.1155/2023/5548684)
Supplement: Supplementary Materials — Table S1: the two answers from ChatGPT that were modified before being sent for grading in their original form and after modification. Table S2: answers to each question from the dieticians and ChatGPT in Dutch and English. Table S3: the grade of each grading component and the average overall grade for the answer to every question for both ChatGPT and the dieticians. Table S4: summary statistics for the grades of the component scientific correctness. Table S5: summary statistics for the grades of the component actionability. Table S6: summary statistics for the grades of the component comprehensibility. Table S7: the p values of the permutation simulations of the test statistic with the mean and the median. [file 5548684.f1.zip › Table S4.docx]

Table S4: Summary statistics for the grades of the component scientific correctness

Summary Statistics for Scientific Correctness

| Question Number |  | Mean | Median | Interquartile Range | Minimum | Maximum |
| --- | --- | --- | --- | --- | --- | --- |
| 1 | Dietician | 6.85 | 7 | 2.00 | 0 | 10 |
| 1 | ChatGPT | 7.44 | 7 | 2.00 | 0 | 10 |
| 2 | Dietician | 7.69 | 8 | 2.75 | 4 | 10 |
| 2 | ChatGPT | 7.46 | 8 | 1.00 | 5 | 9 |
| 3 | Dietician | 7.65 | 8 | 1.50 | 4 | 10 |
| 3 | ChatGPT | 7.46 | 8 | 3.00 | 4 | 10 |
| 4 | Dietician | 7.63 | 8 | 2.00 | 5 | 10 |
| 4 | ChatGPT | 8.39 | 8 | 1.00 | 6 | 10 |
| 5 | Dietician | 6.52 | 6 | 3.00 | 3 | 10 |
| 5 | ChatGPT | 7.26 | 8 | 3.00 | 3 | 10 |
| 6 | Dietician | 7.41 | 7 | 2.50 | 5 | 10 |
| 6 | ChatGPT | 7.80 | 8 | 2.00 | 4 | 10 |
| 7 | Dietician | 7.17 | 7 | 2.50 | 5 | 10 |
| 7 | ChatGPT | 8.00 | 8 | 2.00 | 4 | 10 |
| 8 | Dietician | 6.43 | 6 | 1.50 | 2 | 10 |
| 8 | ChatGPT | 8.33 | 8 | 1.00 | 6 | 10 |
