# Supplementary material for: Comparison of Answers between ChatGPT and Human Dieticians to Common Nutrition Questions
Source: J Nutr Metab. 2023 Nov 7;2023:5548684. doi: 10.1155/2023/5548684 (PMC10645493; doi:10.1155/2023/5548684)
Supplement: Supplementary Materials — Table S1: the two answers from ChatGPT that were modified before being sent for grading in their original form and after modification. Table S2: answers to each question from the dieticians and ChatGPT in Dutch and English. Table S3: the grade of each grading component and the average overall grade for the answer to every question for both ChatGPT and the dieticians. Table S4: summary statistics for the grades of the component scientific correctness. Table S5: summary statistics for the grades of the component actionability. Table S6: summary statistics for the grades of the component comprehensibility. Table S7: the p values of the permutation simulations of the test statistic with the mean and the median. [file 5548684.f1.zip › Table S7.docx]

Table S7: The p-values of the permutation simulations of the test statistic with the mean and the median.

|  | **Overall** | | **Scientific Correctness** | | **Actionability** | | **Comprehensibility** | |
| --- | --- | --- | --- | --- | --- | --- | --- | --- |
|  | **Mean** | **Median** | **Mean** | **Median** | **Mean** | **Median** | **Mean** | **Median** |
| Question 1 | 0.00344 | 0.01243 | 0.03875 | 0.06339 | 0.02149 | 0.54153 | 0.00992 | 0.40591 |
| Question 2 | 0.03118 | 0.11504 | 0.56841 | 0.11578 | 0.00022 | 0.54061 | 0.03057 | 0.18896 |
| Question 3 | 0.67532 | 0.54306 | 0.60639 | 1 | 0.28655 | 1 | 0.22457 | 1 |
| Question 4 | 0.05268 | 0.06453 | 0.02406 | 0.06416 | 0.10513 | 0.28611 | 0.21785 | 1 |
| Question 5 | 0.24663 | 0.18851 | 0.02966 | 0.11406 | 0.83879 | 1 | 0.52821 | 1 |
| Question 6 | 0.0001 | 0.00422 | 0.39515 | 1 | 0.00044 | 0.03038 | <0.0001 | 0.03068 |
| Question 7 | 0.02307 | 0.01237 | 0.0311 | 0.11431 | 0.03579 | 0.18732 | 0.07288 | 0.4052 |
| Question 8 | 0.0001 | 0.00093 | <0.0001 | 0.00097 | 0.00063 | 0.28694 | 0.01683 | 0.28678 |
